# Spatial autocorrelation of environmental factors influencing dengue outbreaks using Moran’s I: A study from Nepal (2020–2023)

**DOI:** 10.1371/journal.pone.0324798

**Published:** 2025-06-04

**Authors:** Roshan Kumar Mahato, Kyaw Min Htike, Kittipong Sornlorm, Alex Bagas Koro, Rajesh Kumar Yadav, Alok Kafle, Vijay Sharma

**Affiliations:** 1 Department of Health Management Innovative Technology, Faculty of Public Health, Khon Kaen University, Khon Kaen, Thailand; 2 Department of Public Health, LA GRANDEE International college, Pokhara University, Nepal; 3 Tropical Medicine, Faculty of Medicine, Khon Kaen University, Thailand; 4 Kathmandu University School of Medical Sciences, Dhulikhel, Nepal; PROPUL Evidence LLP, INDIA

## Abstract

**Background:**

Dengue fever, a mosquito-borne viral infection caused by the dengue virus, has become a significant global public health concern, especially in tropical and subtropical regions. Nepal, with its diverse geography and climate, has witnessed a rapid escalation in dengue cases in recent years, with the highest number of cases and fatalities reported in 2022.

**Objectives:**

This study analyzed the spatial distribution of dengue in Nepal from 2020 to 2023, using Moran’s I spatial statistics to explore the relationship between environmental factors (such as vegetation indices, land surface temperature and precipitation) and dengue incidence.

**Methods:**

By utilizing Geographic Information System (GIS) and spatial analysis techniques, the study seeks to identify high-incidence clusters and examine environmental factors contributing to the spread of dengue.

**Results:**

This study examined dengue incidence in Nepal from 2020 to 2023, uncovering significant variations in disease patterns and their environmental correlations. Dengue cases peaked in 2022 (Moran’s I*;* 0.634, P-value; 0.001) before declining in 2023 (Moran’s I; 0.144, P-value; 0.036), likely due to targeted public health interventions. Spatial analysis revealed no significant patterns in 2020 (Moran’s I; −0.004, P-value; 0.288) and 2021 (Moran’s I; 0.006, P-value; 0.186), however, a focused spatial distribution emerged in 2022 and 2023. Environmental factors showed evolving relationships with dengue transmission: NDVI and LST showed negative correlations in 2020–2021, while NDWI and precipitation shifted from negative to positive correlations over the study period.

**Conclusion:**

The findings showed significant spatial clustering of dengue cases in urban areas with correlations between higher precipitation and increased dengue incidence. These results highlighted the importance of adaptive public health strategies that account for environmental factors.

## Introduction

Dengue fever is a mosquito-borne viral infection caused by the dengue virus, which belongs to the Flaviviridae family and there are four distinct but closely related serotypes of the virus that cause dengue (DENV-1, DENV-2, DENV-3, and DENV-4) [1]. It is transmitted primarily by Aedes spp. mosquitoes, particularly *Aedes aegypti* and, to a lesser extent, *Aedes albopictus* [2]. Dengue fever is prevalent in tropical and subtropical regions around the world, especially in urban and semi-urban areas where these mosquitoes thrive [3]. Each dengue outbreak begins with high mortality and morbidity, leading to a substantial socioeconomic impact. The global incidence of dengue has surged in recent years, as reported cases to the World Health Organization (WHO) escalated from 505,430 in 2000 to a significant 5.2 million in 2019. In 2023, dengue cases reached a peak, affecting more than 80 countries worldwide [3].

Dengue has emerged as a significant and rapidly escalating concern in Nepal. The country has actively engaged in vector surveillance across multiple districts, uncovering the presence of *Aedes aegypti* and *Aedes albopictus* mosquitoes, which serve as vectors for dengue transmission. Nepal has documented the circulation of all four dengue serotypes historically, although, in 2022, DENV-1 and DENV-3 were predominant, with no instances of DENV-4 detected. During the same year, Nepal reported 54,784 confirmed dengue cases and 88 deaths, marking the highest recorded figures in the country’s history. This number exceeded threefold compared to the cases reported in 2019 [4].

The national guidelines on prevention, management, and control of dengue in Nepal emphasize strategies to curb vector-borne diseases. These efforts entail addressing both larval development and adult mosquito populations through environmental management and behavioral changes. Larvicides and biological control are employed against larvae, while space spraying and genetic techniques target adult mosquitoes. Additionally, personal protection measures like mosquito nets and repellents play a crucial role in reducing human-vector contact [5].

Spatial analysis is vital in identifying and managing dengue fever clusters and enhancing the effectiveness of public health interventions. Utilizing Geographic Information Systems (GIS) to map and visualise dengue case distributions facilitates the identification of areas with high incidence rates. Techniques such as spatial autocorrelation [6], kernel density estimation [7], and space-time scan statistics [8,9] help detect and analyse clusters, revealing areas with significant concentrations of cases and recent outbreaks. Additionally, spatial analysis examines environmental factors, like NDVI (Normalized Difference Vegetation Index) [10], NDWI (Normalized Difference Water Index) [11], LSTD (Land Surface Temperature Daytime) [12], LSTN (Land Surface Temperature Nighttime) [13] and precipitation [14] to understand their role in dengue spread. These factors are critical for understanding their influence on dengue transmission dynamics. Continuous monitoring and real-time surveillance facilitate the early detection of new clusters, ensuring prompt and targeted public health responses. By optimizing resource allocation to high-risk areas and evaluating the effectiveness of intervention strategies, spatial analysis contributes to controlling current dengue outbreaks and preventing future ones. This study examined the relationship between environmental factors and dengue incidence in Nepal, addressing the gap in understanding how variables such as vegetation, temperature and precipitation affect dengue transmission. The study hypothesizes that areas with higher vegetation and cooler temperatures will exhibit lower dengue incidence while increased precipitation will correlate with higher incidence rates.

## Materials and methods

### Study area

Nepal, located between India and the Tibet autonomous Region of the People’s Republic of China, spans 147,181 square kilometers, stretching 885 km from east to west and 193 km from north to south. With a population of approximately 29 million, including an absent population of 2.2 million and a growth rate of 0.92% per annum (as per the 2021 census). Nepal lies between 26°22′ and 30°27′ North latitudes and 80°4′ and 88°12′ East longitudes and is administratively divided into 7 provinces and 77 districts, providing structure and governance throughout the country. The country features a diverse topography, from the fertile Gangetic plains to the majestic Himalayas. The Upper Himalaya, covering 15% of the area, includes eight of the world’s highest peaks and attracts trekkers and mountaineers. The Middle Hills and Lower Himalayas, forming 68% of the land, boast a temperate climate and fertile soil, housing the capital city Kathmandu and other key cities. The Tarai Region, occupying 17%, is a fertile agricultural belt with significant wildlife reserves. Nepal experiences four seasons with rapid climate shifts, from the hot Tarai (up to 45°C) to the frigid Himalayas (below −30°C), while Kathmandu enjoys mild temperatures year-round [15].

### Source of data

The Dengue data is extracted from the Nepal Epidemiology and Disease Control Division (EDCD), Ministry of Health and Population (MOHP), Nepal [16].

This study utilized multiple data sources to analyse various environmental and socio-economic factors. Vegetation indices, such as the Normalized Difference Vegetation Index (NDVI) and Normalized Difference Water Index (NDWI), were extracted from the MOD09A1.061 Terra Surface Reflectance 8-Day Global 500m dataset which provides 8-day composites of surface reflectance at a 500-meter spatial resolution [17]. Additionally, Land Surface Temperature (LST) data for day and night were obtained from the same MOD09A1.061 dataset provided by NASA’s Land Processes Distributed Active Archive Centre (LP DAAC) at the USGS EROS Centre [17]. Precipitation data were derived from the CHIRPS Pentad: Climate Hazards Group InfraRed Precipitation With Station Data (Version 2.0 Final) dataset from NASA’s LP DAAC at the USGS EROS Centre [18].

### Statistical analysis

The researchers validated and cleaned the raw dataset to ensure accuracy and reliability. Spatial data from the Moderate Resolution Imaging Spectroradiometer (MODIS) and Climate Hazards InfraRed Precipitation with Station Data (CHIRPS) were integrated into Quantum GIS (QGIS) version 3.36 (Maidenhead) for spatial and non-spatial data processing, generating a shapefile for analysis. Missing data points were addressed using interpolation and regions with incomplete environmental data were excluded to enhance accuracy. Dengue incidence from 2020 to 2023 was visualized in QGIS to identify spatial patterns and potential high-incidence clusters. Spatial autocorrelation was assessed using Moran’s I and Local Indicators of Spatial Association (LISA) in GeoDa version 1.22.

### Univariate and bivariate analysis

The initial step in our analysis involved utilizing Global and Local Moran’s I statistics to identify spatial autocorrelation in the incidence of Dengue. Global Moran’s I was employed to detect broad trends across the entire country, while Local Moran’s I was used to pinpoint clusters or hotspots at the local level. Global Moran’s I was calculated using the equation:


$$I=\frac{n}{S_{0}}\text{ * }\frac{Z_{i}Z_{j}W_{ij}}{Z_{i}^{2}}$$ (Eq.1)

and Local Moran’s I was computed using the equation:


$$I_{1}=\frac{Z_{i}}{S_{1}}\text{ * }Z_{j}W_{ij}$$ (Eq.2)

where *n* represents the total number of regions, *S0* denotes the sum of all spatial weights, *Z* indicates the deviation of the variable from its mean, *S1* is the sum of all squared deviations, and *Wij* is the spatial weight between regions *I* and *j*.

For this study, spatial autocorrelation was assessed using Local Indicators of Spatial Association (LISA), a category of statistics that includes various measures to detect different types of spatial patterns, including Moran’s I. LISA was applied to assess global spatial autocorrelation in the incidence of Dengue and its associated factors. Specifically, this study utilized Moran’s I within the LISA framework to determine whether individual regions were part of a spatial cluster with similar Dengue incidence values (i.e., high-high or low-low clusters) or to identify outliers (i.e., high-low or low-high clusters). A weight matrix with three clusters of K- nearest neighbours was used for univariate and bivariate analysis. This study employed 999 permutations to evaluate how sensitive the significant locations were to the number of permutations, with a significance level of p < 0.05.

### Ethical considerations

The Khon Kaen University Ethics Committee for Human Research (KKUEC) has authorized this study on the basis of exemption for ethical approval (Reference Number: HE 672162).

## Results

### Annual trend of Dengue incidence in Nepal (2020–2023)

**Fig 1:**
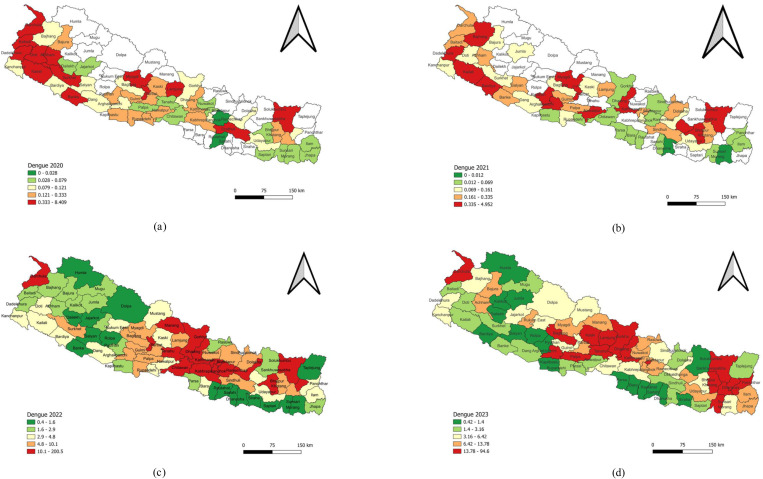
Annual trend of Dengue Incidence in Nepal (2020–2023). (a) Incidence of Dengue in 2020. (b) Incidence of Dengue in 2021. (c) Incidence of Dengue in 2022. (d) Incidence of Dengue in 2023.

The Dengue incidence in 2020 and 2021 was not spatially significant, while those in 2022 and 2023 showed spatial significance. In 2020, Dengue incidence was 0.19 per 10,000 population. This decreased slightly to 0.16 per 10,000 in 2021. However, in 2022, incidence rose sharply to 17.60 per 10,000 population. The highest prevalence was recorded in Lalitpur, with 200.54 cases per 10,000 population, while the lowest was in Dhanusha, with 0.38 cases per 10,000 population. A quantile map with equal counts highlighted the high incidence clusters (10.1–200.5 cases per 10,000 population) in 16 districts, including Lalitpur, Bhaktapur, Makawanpur, Kathmandu, Dhading, Chitawan, Kabhrepalanchok, Darchula, Terhathum, Parbat, Sankhuwasabha, Khotang, Ramechhap, Manang, Gorkha, and Tanahu. In 2023, the overall Dengue incidence was 9.35 per 10,000 population. The highest prevalence was found in Tanahu, with 94.60 cases per 10,000 population, while the lowest was in Jumla, with 0.42 cases per 10,000 population. The quantile map indicated the high incidence clusters (13.79–94.6 cases per 10,000 population) in 16 districts, including Tanahu, Dhading, Sankhuwasabha, Sunsari, Panchthar, Bhojpur, Dhankuta, Terhathum, Kaski, Darchula, Palpa, Bhaktapur, Baglung, Lamjung, Gorkha, and Arghakhanchi (Table 1 and Fig 1).

**Table 1:** Annual trend of Dengue Incidence in Nepal (2020–2023).

Year	Incidence Rate (Cases/10,000)	Z Score	P-value
2020	0.19	0.32	0.288
2021	0.16	0.37	0.186
2022	17.60	9.82	0.001
2023	9.35	2.13	0.036

### Univariate Moran’ I analysis for Dengue incidence 2020–2023

**Fig 2:**
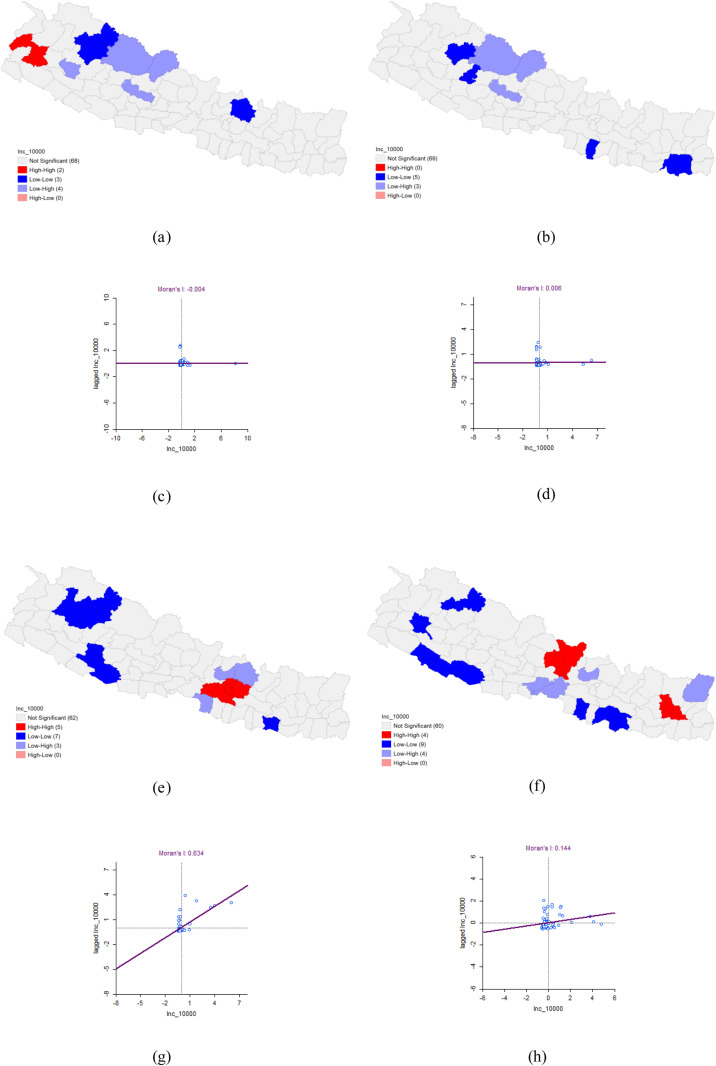
Univariate Moran’ I analysis for Dengue incidence 2020–2023. (a) LISA map of incidence of Dengue in 2020. (b) LISA map of incidence of Dengue in 2021. (c) Moran’s I scatter plot of incidence of Dengue in 2020. (d) Moran’s I scatter plot of incidence of Dengue in 2021. (e) LISA map of incidence of Dengue in 2022. (f) LISA map of incidence of Dengue in 2023. (g) Moran’s I scatter plot of incidence of Dengue in 2022. (h) Moran’s I scatter plot of incidence of Dengue in 2023.

The univariate Moran’s I analysis of annual Dengue incidence in 2020 revealed a negative spatial autocorrelation, with a Moran’s I value of −0.004, which was not statistically significant based on 999 permutations. In 2021, the univariate Moran’s I analysis indicated a positive spatial autocorrelation with a Moran’s I value of 0.006, which also lacked statistical significance according to 999 permutations. For 2022, the univariate Moran’s I analysis showed a positive spatial autocorrelation with a Moran’s I value of 0.634, which was statistically significant (p < 0.05) based on 999 permutations. High-high clusters of Dengue incidence were primarily located in the capital region of Nepal, including Kathmandu, Lalitpur, Bhaktapur, Kabhrepalanchok, and Makawanpur, while low-low clusters were found in Siraha, Dang, Salyan, Bajura, Mugu, Jumla, and Kalikot. In 2023, the univariate Moran’s I analysis also showed a positive spatial autocorrelation with a Moran’s I value of 0.144, which was statistically significant (p < 0.05) based on 999 permutations. High-high clusters of Dengue incidence were identified in Lamjung, Gorkha, Bhojpur, and Dhankuta, while low-low clusters were observed in Achham, Bardiya, Banke, Dang, Mugu, Bara, Sarlahi, Mahottari, and Dhanusha (Table 2 and Fig 2).

**Table 2:** Univariate Moran’ I analysis for Dengue incidence 2020–2023.

Dengue cluster	Moran’s I	LISA				P-value
		HH	HL	LH	LL	
2020	−0.004	Baitadi* Doti*		Dailekh* Dolpa* Mustang* Baglung**	Mugu* Jumla* Sindhupal-chok*	0.05* 0.01** 0.001***
2021	0.006			Dolpa* Mustang* Baglung**	Jumla* Rukum West* Rautahat* Sunsari* Morang*	
2022	0.634	Kathmandu*** Lalitpur*** Bhaktapur** Kabhrepalan-chok*** Makawanpur***		Nuwakot** Sindhupal-chok* Parsa*	Siraha* Dang* Salyan* Bajura* Mugu* Jumla* Kalikot*	
2023	0.144	Lamjung* Gorkha* Bhojpur* Dhankuta*		Nuwakot* Nawalpur* Chitawan* Taplejung**	Achham* Bardiya* Banke* Dang* Mugu** Bara* Sarlahi** Mahottari* Dhanusha*	

### Univariate analysis of environmental variables of Nepal

#### Distribution of Normalized Difference Vegetation Index (NDVI) 2020–2023.

**Fig 3:**
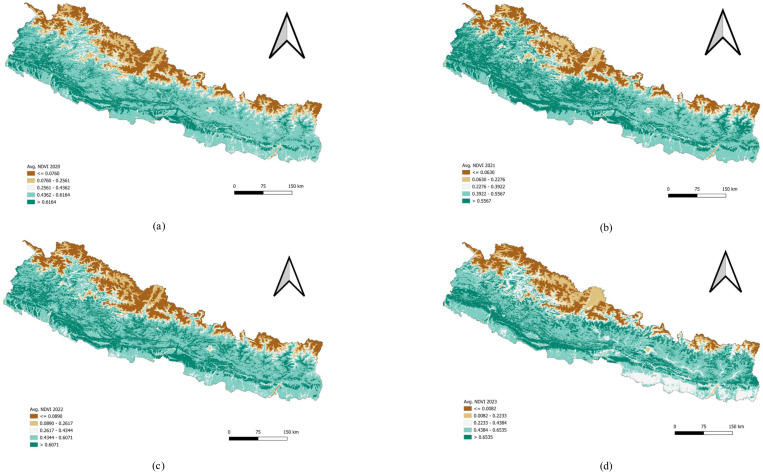
Univariate analysis of Normalized Difference Vegetation Index (NDVI) 2020–2023. (a) Average NDVI 2020. (b) Average NDVI 2021. (c) Average NDVI 2022. (d) Average NDVI 2023. Distribution of Normalized Difference Water Index (NDWI).

High NDVI values are generally found in areas with dense vegetation, such as forests and well-irrigated agricultural lands in the mid-hills and Terai. Moderate NDVI values are seen in regions with less dense vegetation, while low NDVI values are typical in the high-altitude Himalayan regions, urban areas, and barren lands. This variation in NDVI reflects Nepal’s diverse climate, topography, and land use patterns, ranging from fertile plains to rugged mountains (Fig 3).

**Fig 4:**
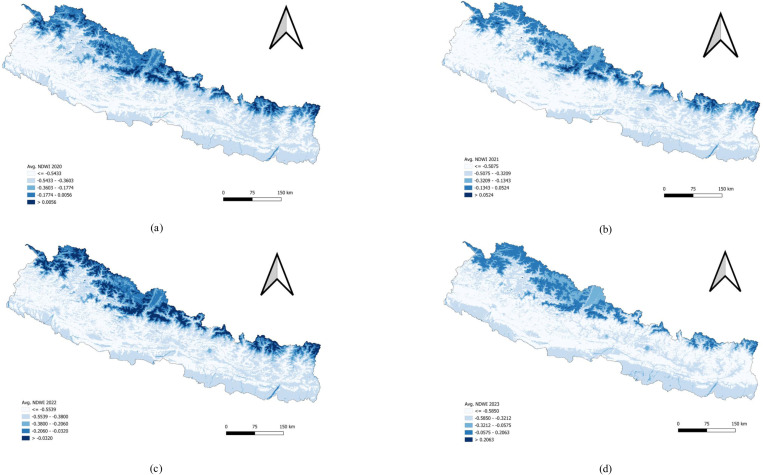
Univariate analysis of Normalized Difference Water Index (NDWI) 2020–2023. (a) Average NDWI 2020. (b) Average NDWI 2021. (c) Average NDWI 2022. (d) Average NDWI 2023. Pattern of Land Surface Temperature Day (LSTD).

Regions with high NDWI values likely represent areas with higher water content, such as rivers, lakes, or regions with dense vegetation. These areas are predominantly found in the northern mountainous regions, which may include glaciated areas and perennial rivers. Areas with moderate NDWI values indicate moderate water content, potentially corresponding to regions with a mix of vegetation and water bodies or those that receive moderate rainfall. The regions with the lowest NDWI values suggest low water content, likely representing dry areas, barren lands, or regions with sparse vegetation. The southern plains (Terai) and parts of the middle hills generally exhibit these lower NDWI values (Fig 4).

**Fig 5:**
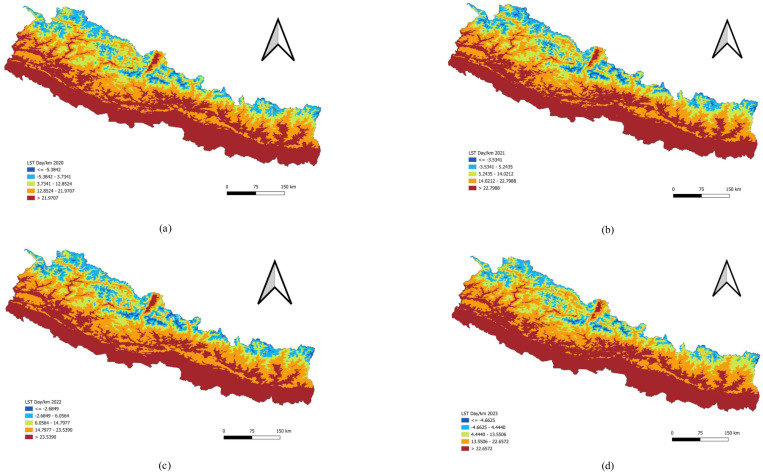
Univariate analysis of Land Surface Temperature Day (LSTD) 2020–2023. (a) LSTD per kilometer 2020. (b) LSTD per kilometer 2021. (c) LSTD per kilometer 2022. (d) LSTD per kilometer 2023. Pattern of Land Surface Temperature Night (LSTN).

The Himalayan region, which includes the higher mountainous and hilly areas of Nepal, experiences the lowest temperatures. The middle hills show moderately high temperatures, while the western Terai region records the highest daytime land surface temperatures. In general, daytime temperatures (LSTD) are elevated in the southern plains and decrease in the mountainous regions (Fig 5).

**Fig 6:**
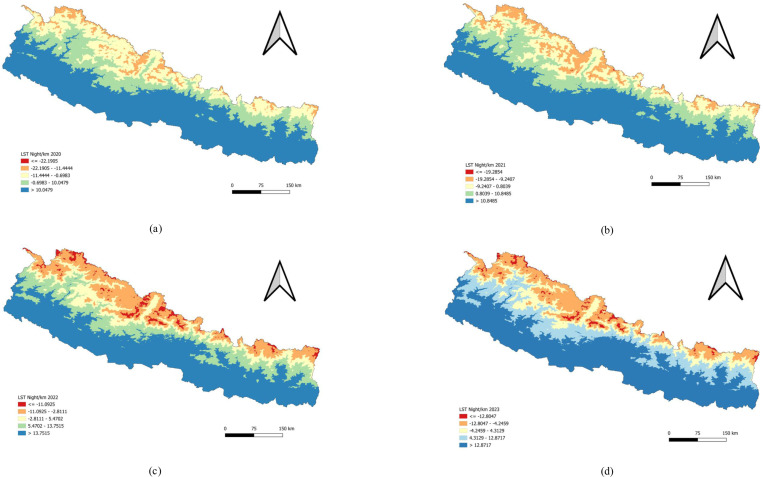
Univariate analysis of Land Surface Temperature Night (LSTN) 2020–2023. (a) LSTN per kilometer 2020. (b) LSTN per kilometer 2021. (c) LSTN per kilometer 2022. (d) LSTN per kilometer 2023. **Distribution of Precipitation.**

At night, regions with the lowest land surface temperatures are found in the high-altitude and hilly areas, where temperatures drop significantly. The middle hills experience moderately cold temperatures at night, while the lower, southern regions of Nepal tend to have warmer nighttime temperatures. Nighttime temperatures (LSTN) are notably lower in the mountains, with some areas becoming very cold, whereas the southern regions stay relatively warmer even during the night (Fig 6).

**Fig 7:**
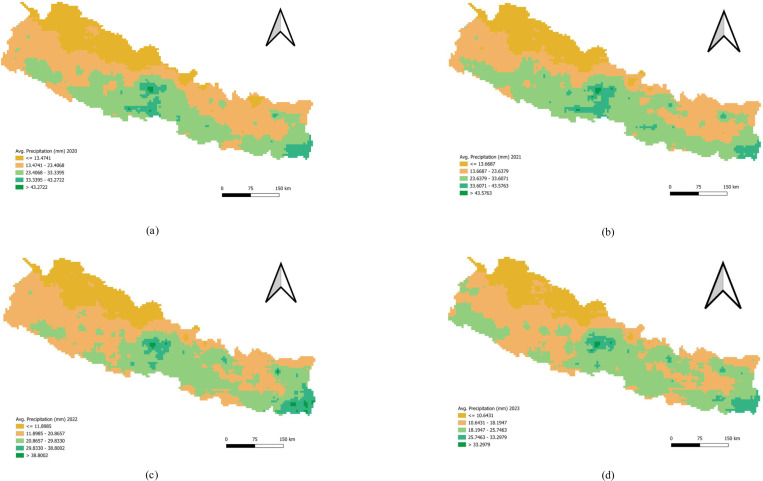
Univariate analysis of Precipitation 2020–2023. (a) Average Precipitation (mm) in 2020. (b) Average Precipitation (mm) in 2021. (c) Average Precipitation (mm) in 2022. (d) Average Precipitation (mm) in 2023 **Bi** Bivariate Analysis of NDVI and Dengue Incidence.

Nepal’s precipitation patterns are shaped by its diverse geography, with high rainfall in the central and eastern mid-hills, moderate rainfall in the western mid-hills and Terai, and low rainfall in the Trans-Himalayan and rain-shadow regions (Fig 7).

In 2020, the bivariate LISA analysis revealed a statistically significant negative correlation between the Normalized Difference Vegetation Index (NDVI) and Dengue incidence, with Moran’s I value of −0.211. This analysis identified high-high clusters in Baitadi, Doti, Dailekh, and Baglung districts, where high NDVI values coincided with high Dengue incidence, and these areas were surrounded by districts with similarly high values. Conversely, low-low clusters were detected in Mugu, Jumla, and Sinduhupalchok districts, where both NDVI and Dengue incidence were low, surrounded by districts with similarly low values.

**Fig 8:**
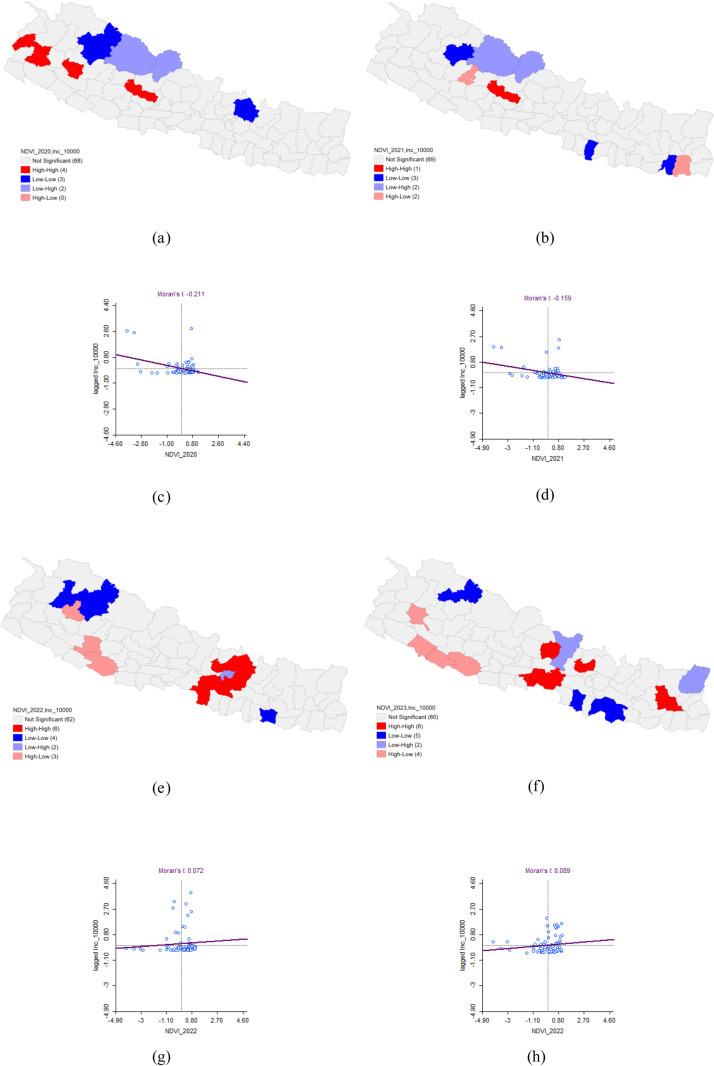
Bivariate Analysis of NDVI and Dengue Incidence 2020–2023. (a) LISA map of NDVI and Dengue incidence in 2020. (b) LISA map of NDVI and Dengue incidence in 2021. (c) Moran’s I scatter plot of NDVI and Dengue incidence in 2020. (d) Moran’s I scatter plot of NDVI and Dengue incidence in 2021. (e) LISA map of NDVI and Dengue incidence in 2022. (f) LISA map of NDVI and Dengue incidence in 2023. (g) Moran’s I scatter plot of NDVI and Dengue incidence in 2022. (h) Moran’s I scatter plot of NDVI and Dengue incidence in 2023. Bivariate analysis of NDWI and Dengue Incidence.

In 2021, the negative correlation between NDVI and Dengue incidence persisted, with Moran’s I value of −0.159. The LISA analysis identified a high-high cluster in Baglung district, where high NDVI and high Dengue incidence were concentrated, surrounded by three districts with similarly high values. Additionally, low-low clusters were found in Jumla, Rautahat, and Sunsari districts, where both NDVI and Dengue incidence were low, with neighboring districts also exhibiting low values. For 2022 and 2023, the spatial analysis did not reveal any significant patterns (Table 3 and Fig 8).

**Table 3:** Bivariate Analysis of NDVI and Dengue Incidence.

Dengue cluster	Moran’s I	LISA				P-value
		HH	HL	LH	LL	
2020	−0.211	Baitadi* Doti* Dailekh* Baglung**		Dolpa* Mustang*	Mugu* Jumla* Sinduhupalchok*	0.05* 0.01** 0.001***
2021	−0.159	Baglung**	Rukum West*** Morang*	Dolpa* Mustang*	Jumla* Rautahat* Sunsari*	
2022	0.072	Parsa* Makawanpur*** Nuwakot** Kabhrepa-lanchok *** Sindhupal- chok* Lalitpur***	Kalikot* Dang* Salyan*	Kathmandu*** Bhaktapur**	Bajura* Mugu* Jumla* Siraha*	
2023	0.089	Lamjung* Nuwakot* Nawalpur* Chitawan* Bhojpur* Dhankuta*	Achham* Bardiya* Banke* Dang*	Gorkha* Taplejung**	Mugu** Bara* Sarlahi** Mahottari* Dhanusha*	

In 2020, the bivariate LISA analysis revealed a statistically significant negative correlation between the Normalized Difference Water Index (NDWI) and Dengue incidence, with Moran’s I value of −0.177. This analysis identified high-high clusters in Doti, Dailekh, and Baglung districts, where high NDWI values were associated with high Dengue incidence, and these areas were surrounded by districts with similarly high values. Conversely, low-low clusters were detected in Mugu and Jumla districts, where both NDWI and Dengue incidence were low, surrounded by districts with similarly low values.

In 2021, a statistically significant negative correlation between NDWI and Dengue incidence persisted (Moran’s I = −0.128). The analysis identified high-high clusters in Baglung district, where high NDWI and high Dengue incidence were concentrated, with surrounding districts also exhibiting high values. Low-low clusters were observed in Jumla, Rukum West, Rautahat, Sunsari, and Morang districts, where both NDWI and Dengue incidence were low, surrounded by districts with similarly low values.

**Fig 9:**
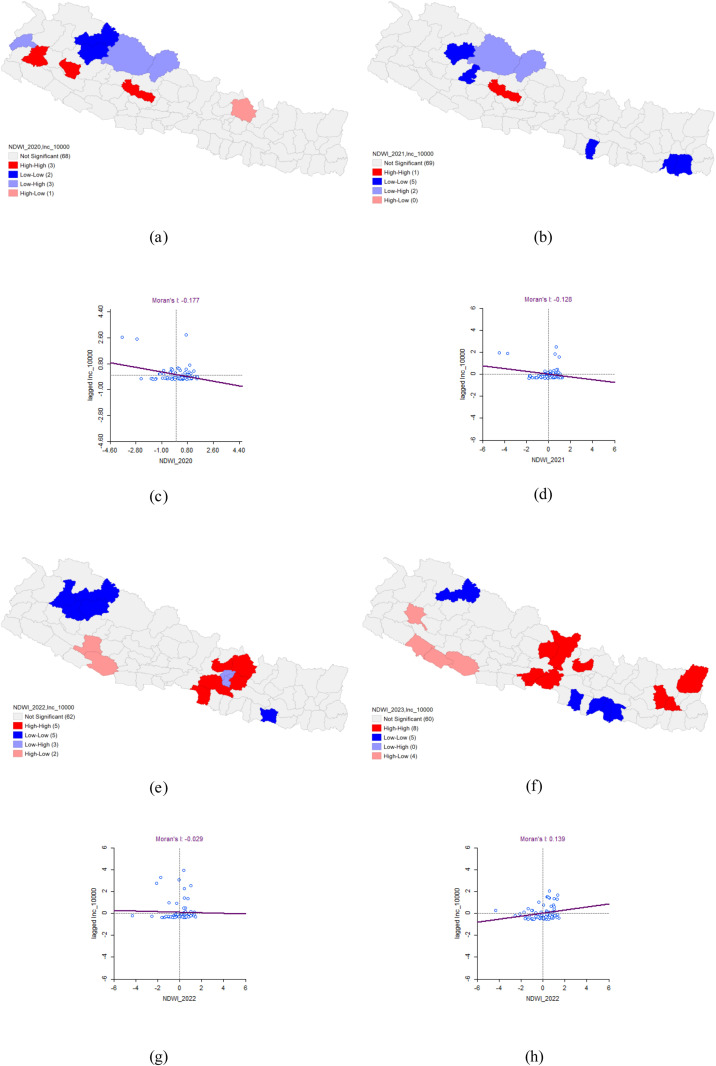
Bivariate Analysis of NDWI and Dengue Incidence 2020–2023. (a) LISA map of NDWI and Dengue incidence in 2020. (b) LISA map of NDWI and Dengue incidence in 2021. (c) Moran’s I scatter plot of NDWI and Dengue incidence in 2020. (d) Moran’s I scatter plot of NDWI and Dengue incidence in 2021. (e) LISA map of NDWI and Dengue incidence in 2022. (f) LISA map of NDWI and Dengue incidence in 2023. (g) Moran’s I scatter plot of NDWI and Dengue incidence in 2022. (h) Moran’s I scatter plot of NDWI and Dengue incidence in 2023.

The spatial analysis for 2022 did not reveal any significant patterns. In 2023, the spatial autocorrelation between NDWI and Dengue incidence showed a positive correlation (Moran’s I = 0.139), indicating that both variables exhibited similar distribution patterns. The LISA analysis identified eight high-high clusters—Gorkha, Nuwakot, Lamjung, Chitawan, Dhankuta, Bhojpur, Taplejung, and Nawalpur districts—where high NDWI and high Dengue incidence were concentrated, with surrounding districts also showing high values. Additionally, five low-low clusters were found in Mugu, Bara, Mahottari, Dhanusha, and Sarlahi districts, where both NDWI and Dengue incidence were low, with adjacent districts exhibiting similarly low values (Table 4 and Fig 9).

**Table 4:** Bivariate Analysis of NDWI and Dengue Incidence.

Dengue cluster	Moran’s I	LISA				P-value
		HH	HL	LH	LL	
2020	−0.177	Doti* Dailekh* Baglung**	Sinduhupalchok*	Dolpa* Mustang* Baitadi*	Mugu* Jumla*	0.05* 0.01** 0.001***
2021	−0.128	Baglung**		Dolpa* Mustang*	Jumla* Rukum West* Rautahat* Sunsari* Morang*	
2022	−0.029	Parsa* Makawanpur*** Nuwakot** Kabhrepalan-chok*** Sindhupal- chok*	Dang* Salyan*	Kathmandu*** Lalitpur*** Bhaktapur**	Bajura* Mugu* Jumla* Kalikot* Siraha*	
2023	0.139	Gorkha* Nuwakot* Lamjung* Chitawan* Dhankuta* Bhojpur* Taplejung** Nawalpur*	Bardiya* Banke* Dang* Achham*		Mugu** Bara* Mahottari* Dhanusha* Sarlahi**	

### Bivariate analysis of LSTD and Dengue incidence

In 2020, the bivariate LISA analysis revealed a statistically significant negative correlation between Land Surface Temperature during the Day (LSTD) and Dengue incidence, with Moran’s I value of −0.141. This analysis identified high-high clusters in Doti, Dailekh, and Baitadi districts, where high LSTD values were associated with a high incidence of Dengue, and these areas were surrounded by districts with similarly high values. Conversely, low-low clusters were observed in Mugu, Jumla, and Sindhupalchok districts, where both LSTD values and Dengue incidence were low, with neighboring districts also exhibiting low values.

**Fig 10:**
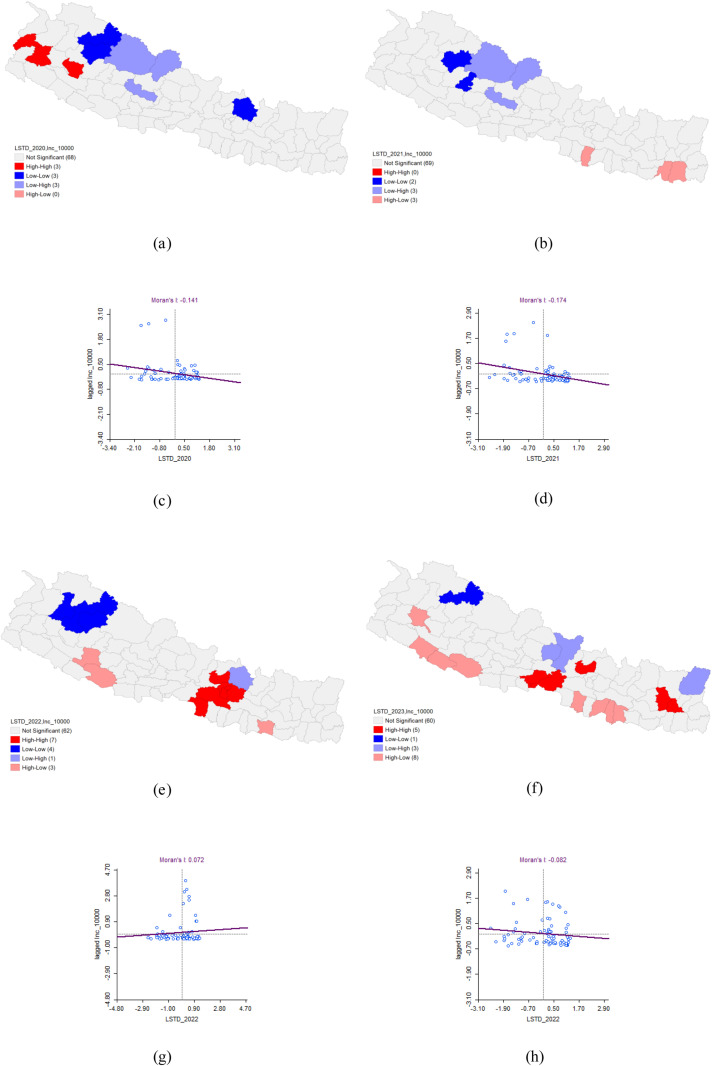
Bivariate Analysis of LSTD and Dengue Incidence 2020–2023. (a) LISA map of LSTD and Dengue incidence in 2020. (b) LISA map of LSTD and Dengue incidence in 2021. (c) Moran’s I scatter plot of LSTD and Dengue incidence in 2020. (d) Moran’s I scatter plot of LSTD and Dengue incidence in 2021. (e) LISA map of LSTD and Dengue incidence in 2022. (f) LISA map of LSTD and Dengue incidence in 2023. (g) Moran’s I scatter plot of LSTD and Dengue incidence in 2022. (h) Moran’s I scatter plot of LSTD and Dengue incidence in 2023.

The bivariate LISA revealed a statistically significant negative correlation between LSTD and the incidence of Dengue in 2021 (Moran’s I = −0.174). LISA indicated that there were no districts with a significant concentration of LSTD and a high incidence of Dengue forming High-High clusters. However, it did show areas with varied patterns, but none reached the threshold for being identified as significant Hot-spot or High-High clusters. In contrast, LISA analysis showed clusters of a district with a low LSTD value and Dengue patients with low values in the surrounding 3 districts (Cold-spot or low-low clusters). There were 2 low-low clusters found in Jumla and Rukum West districts. For 2022 and 2023, the spatial analysis did not reveal any significant patterns (Table 5 and Fig 10).

**Table 5:** Bivariate Analysis of LSTD and Dengue Incidence.

Dengue cluster	Moran’s I	LISA				P-value
		HH	HL	LH	LL	
2020	−0.141	Doti* Dailekh* Baitadi*		Dolpa* Mustang* Baglung**	Mugu* Jumla* Sinduhupal-chok*	0.05* 0.01** 0.001***
2021	−0.174		Rautahat* Sunsari* Morang*	Dolpa* Mustang* Baglung**	Jumla* Rukum West*	
2022	0.072	Kathmandu*** Lalitpur*** Bhaktapur** Parsa* Makawanpur*** Nuwakot** Kabhrepalan-chok***	Siraha* Dang* Salyan*	Sindhupal- chok*	Bajura* Mugu* Jumla* Kalikot*	
2023	−0.082	Dhankuta* Bhojpur* Nuwakot* Nawalpur* Chitawan*	Bardiya* Banke* Dang* Achham* Bara* Mahottari* Dhanusha* Sarlahi**	Gorkha* Lamjung* Taplejung**	Mugu**	

### Bivariate analysis of LSTN and Dengue incidence

The bivariate LISA revealed a statistically significant negative correlation between LSTN and the incidence of Dengue in 2020 (Moran’s I = −0.187). LISA indicated areas with a concentration of LSTN and a high incidence of Dengue with high values in the surrounding 3 districts (Hot-spot or High-High cluster) in Doti, Dailekh and Baitadi. In contrast, LISA analysis showed clusters of a district with a low LSTN value and Dengue patients with low values in the surrounding 3 districts (Cold-spot or low-low clusters). There were 3 low-low clusters found in Mugu, Jumla and Sinduhupalchok districts.

**Fig 11:**
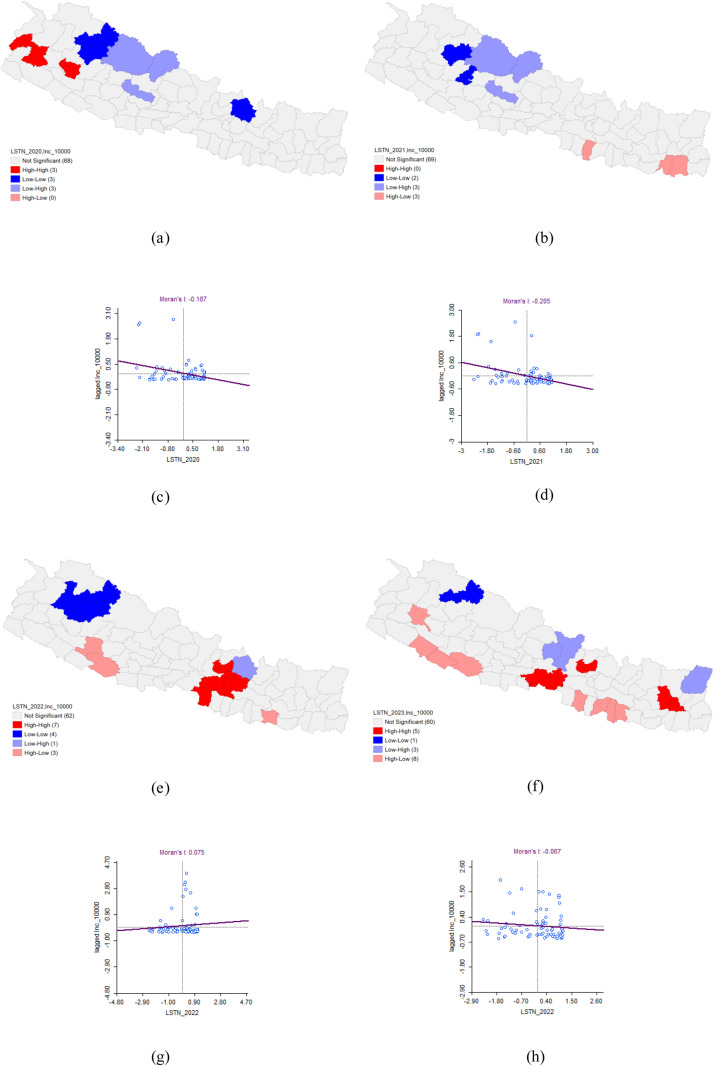
Bivariate Analysis of LSTN and Dengue Incidence 2020–2023. (a) LISA map of LSTN and Dengue incidence in 2020. (b) LISA map of LSTN and Dengue incidence in 2021. (c) Moran’s I scatter plot of LSTN and Dengue incidence in 2020. (d) Moran’s I scatter plot of LSTN and Dengue incidence in 2021. (e) LISA map of LSTN and Dengue incidence in 2022. (f) LISA map of LSTN and Dengue incidence in 2023. (g) Moran’s I scatter plot of LSTN and Dengue incidence in 2022. (h) Moran’s I scatter plot of LSTN and Dengue incidence in 2023.

In 2021, the analysis again found a statistically significant negative correlation between LSTN and Dengue incidence (Moran’s I = −0.205). This year, no districts were identified as significant high-high clusters where high LSTN and high Dengue incidence were concentrated. However, the analysis did reveal two low-low clusters in Jumla and Rukum West districts, where both LSTN and Dengue incidence were low, surrounded by districts with similarly low values. For 2022 and 2023, the spatial analysis did not reveal any significant patterns (Table 6 and Fig 11).

**Table 6:** Bivariate Analysis of LSTN and Dengue Incidence.

Dengue cluster	Moran’s I	LISA				P-value
		HH	HL	LH	LL	
2020	−0.187	Doti* Dailekh* Baitadi*		Dolpa* Mustang* Baglung**	Mugu* Jumla* Sinduhupal-chok*	0.05* 0.01** 0.001***
2021	−0.205		Rautahat* Sunsari* Morang*	Dolpa* Mustang* Baglung**	Jumla* Rukum West*	
2022	0.075	Kathmandu*** Lalitpur*** Bhaktapur** Parsa* Makawanpur*** Nuwakot** Kabhrepalan-chok***	Siraha* Dang* Salyan*	Sindhupal- chok*	Bajura* Mugu* Jumla* Kalikot*	
2023	−0.067	Dhankuta* Bhojpur* Nuwakot* Nawalpur* Chitawan*	Bardiya* Banke* Dang* Achham* Bara* Mahottari* Dhanusha* Sarlahi**	Gorkha* Lamjung* Taplejung**	Mugu**	

### Bivariate analysis of precipitation and Dengue incidence

In 2020, the bivariate LISA analysis revealed a statistically significant negative correlation between precipitation and Dengue incidence, with Moran’s I value of −0.183. This analysis identified a high-high cluster in Baglung district, where both high precipitation and high Dengue incidence were concentrated, surrounded by three neighboring districts with similarly high values. Conversely, low-low clusters were detected in Mugu, Jumla, and Sindhupalchok districts, where both precipitation and Dengue incidence were low, with surrounding districts also exhibiting low values.

In 2021, a similar negative correlation was observed, with Moran’s I value of −0.159. The analysis again highlighted a high-high cluster in Baglung district, marked by high precipitation and high Dengue incidence, surrounded by three districts with comparable high values. Low-low clusters were identified in Jumla and Rukum West districts, where both precipitation and Dengue incidence were low, along with neighboring districts showing low values.

By 2022, the spatial autocorrelation shifted to a positive correlation (Moran’s I = 0.154), indicating that high and low values of precipitation and Dengue incidence were more aligned. The analysis revealed eight high-high clusters, including Kathmandu, Lalitpur, Bhaktapur, Parsa, Makawanpur, Sindhupalchok, Nuwakot, and Kabhrepalanchok districts, surrounded by similarly high-value districts. Additionally, five low-low clusters were found in Bajura, Mugu, Jumla, Kalikot, and Salyan, with adjacent districts also showing low values.

**Fig 12:**
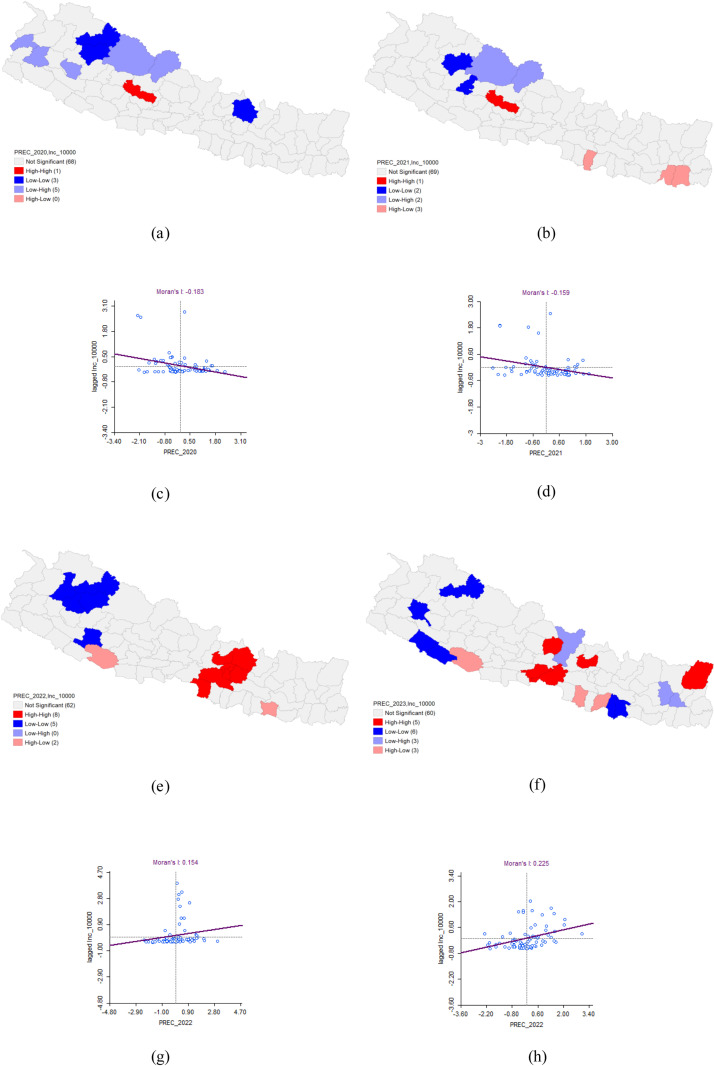
Bivariate Analysis of precipitation and Dengue Incidence 2020–2023. (a) LISA map of precipitation and Dengue incidence in 2020. (b) LISA map of precipitation and Dengue incidence in 2021. (c) Moran’s I scatter plot of precipitation and Dengue incidence in 2020. (d) Moran’s I scatter plot of precipitation and Dengue incidence in 2021. (e) LISA map of precipitation and Dengue incidence in 2022. (f) LISA map of precipitation and Dengue incidence in 2023. (g) Moran’s I scatter plot of precipitation and Dengue incidence in 2022. (h) Moran’s I scatter plot of precipitation and Dengue incidence in 2023.

In 2023, the spatial autocorrelation remained positive, with a Moran’s I value of 0.225. This year’s analysis identified five high-high clusters in Taplejung, Nuwakot, Lamjung, Chitawan, and Nawalpur districts, surrounded by districts with high values. Six low-low clusters were also noted in Bardiya, Banke, Achham, Mugu, Mahottari, and Dhanusha, with neighboring districts exhibiting similarly low values (Table 7 and Fig 12).

**Table 7:** Bivariate Analysis of Precipitation and Dengue Incidence.

Dengue cluster	Moran’s I	LISA				P-value
		HH	HL	LH	LL	
2020	−0.183	Baglung**		Dolpa* Mustang* Doti* Dailekh* Baitadi*	Mugu* Jumla* Sinduhu-palchok*	0.05* 0.01** 0.001***
2021	−0.159	Baglung**	Rautahat* Sunsari* Morang*	Dolpa* Mustang*	Jumla* Rukum West*	
2022	0.154	Kathmandu*** Lalitpur*** Bhaktapur** Parsa* Makawanpur*** Sindhupal- chok* Nuwakot** Kabhrepalan-chok***	Siraha* Dang*		Bajura* Mugu* Jumla* Kalikot* Salyan*	
2023	0.225	Taplejung** Nuwakot* Lamjung* Chitawan* Nawalpur*	Dang* Sarlahi** Bara*	Gorkha* Dhankuta* Bhojpur*	Bardiya* Banke* Achham* Mugu** Mahottari* Dhanusha*	

## Discussion

### Trends of dengue cases in Nepal

This study analysed the incidence of dengue in Nepal from 2020 to 2023. The results indicated incidence rates of 0.185 per 10,000 population in 2020, 0.163 per 10,000 population in 2021, 17.6 per 10,000 population in 2022, and 9.35 per 10,000 population in 2023. The incidence rate increased significantly in 2022, followed by a marked decline in 2023. This decline in 2023 may be attributed to specific interventions implemented in 2022. The MoHP and local health authorities intensified vector control efforts, including large-scale fogging operations, indoor residual spraying, and larval source reduction campaigns. Additionally, public health awareness campaigns on dengue prevention and early symptom recognition were expanded through community engagement and social media platforms [19]. Enhanced surveillance and case detection efforts in 2022 also played a role in reducing the disease burden by enabling a more rapid response to outbreaks [20]. While these interventions contributed to the decline in cases, other factors such as climate variability and human mobility may have also influenced transmission dynamics [21].

An important factor influencing the trends observed in this study is the impact of the SARS-CoV-2 pandemic on dengue transmission [22]. Nepal experienced significant seasonal waves of COVID-19 infections before January 2022, leading to nationwide lockdowns, restricted mobility, and an overwhelmed healthcare system. These restrictive measures likely reduced dengue transmission in 2020 and 2021 by limiting human movement and exposure to mosquito breeding sites in urban and peri-urban areas [23]. The global pandemic may have also impacted surveillance and reporting, leading to potential underreporting of dengue cases during this period [24]. However, as COVID-19 cases declined in the second quarter of 2022, lockdowns were lifted, and urban activities resumed, resulting in increased dengue transmission [25]. This is reflected in the sharp rise in dengue incidence in 2022 and the emergence of significant spatial clustering, as indicated by the Moran’s I values becoming positive and statistically significant from 2022 onwards. Our findings align with a study from Indonesia, where dengue incidence was 4 per 10,000 in 2020 and 2.7 per 10,000 in 2021, following a similar trend [26]. Meanwhile, dengue cases in Timor-Leste fluctuated, with the highest incidence rate (IR) of 2.94 per 1,000 population in 2020 and the lowest IR of 0.00 per 1,000 population in 2021 [27]. In contrast, a 2023 study in Bangladesh reported a significant surge in dengue cases and deaths, with cases increasing five-fold and deaths rising nearly six-fold compared to 2022 [28].

### Geospatial distribution of dengue cases

This study examined Nepal’s geospatial distribution of dengue cases from 2020 to 2023. The distribution was not spatially significant in 2020 and 2021. In 2020, the incidence of dengue had a negative Moran’s I value, while in 2021, it had a positive Moran’s I value, both of which were not spatially significant. However, in 2022, a positive correlation was observed, with a Moran’s I value of 0.634 and a z-score of 9.82 (p < 0.001). In 2023, Moran’s I value was 0.144 with a z-score of 2.13, indicating a positive correlation with spatially significant. Our findings were consistent with previous studies conducted in Ecuador [29], Malaysia [30], Philippines [31] and Indonesia [32], which also reported significant spatial patterns in dengue incidence. In contrast, a study from Brazil noted significant positive spatial autocorrelation in most study years, except for 2011 and 2017, which were characterized by low dengue incidence in Belo Horizonte. The study highlighted those six years of the series exhibited spatial autocorrelation values exceeding 45%. Specifically, the study found a positive spatial correlation between dengue incidence and OPI (Open Public Infrastructure), with the highest Moran’s I values observed in 2010 and 2013. Conversely, 2016 showed a negative Moran’s I value, indicating areas with high dengue incidence but low OPI or vice versa [6]. These insights underscored the complex spatial dynamics of dengue transmission, influenced by local factors and infrastructure, necessitating tailored interventions and surveillance strategies in affected regions.

### Environmental factors associated with Dengue Incidence

Our study identified a significant negative correlation between the Normalized Difference Vegetation Index (NDVI) and dengue incidence in Nepal from 2020 to 2023. Moran’s, I value of −0.211 in 2020 and −0.159 in 2021 revealed a strong spatial autocorrelation, indicating that areas with more robust and dense vegetation were associated with higher dengue transmission rates. However, the data from 2022 and 2023 did not show significant spatial patterns, suggesting variability in the factors that influenced dengue transmission. The dense and healthy vegetation likely provided ideal microhabitats for mosquito breeding, particularly for *Aedes aegypti* and *Aedes albopictus*, the primary dengue vectors. The vegetation offered shade, moisture, and organic matter, which were favourable for mosquito larvae development. Furthermore, the vegetation might have altered the local microclimate, creating conditions that supported mosquito survival and extended the transmission season. The absence of spatial significance in 2022 and 2023 implied that other environmental or socio-economic factors might have played a more prominent role in those years. Factors such as changes in land use, urbanization, climate fluctuations, or public health interventions could have impacted the NDVI-dengue relationship, emphasizing the complexity of dengue ecology. These findings highlighted the importance of incorporating vegetation indices along with other environmental and demographic factors in predictive models to enhance dengue risk assessments and develop more effective intervention strategies.

Our findings are consistent with other studies conducted in China and Bhutan. The study from China, employing a generalized additive model (GAM), and the study from Bhutan, using Bayesian analysis with zero-inflated Poisson (ZIP) regression, both reported a negative relationship between the risk of dengue and NDVI [10,33]. However, these findings are contrast with previous studies. For instance, a study conducted in Brazil using remote sensing indicated that NDVI is a reliable predictor of dengue incidence and serves as a leading indicator up to five weeks in advance [34]. Similarly, a study from Indonesia showed that an increase in NDVI with a one-month lag was associated with a 3.07-fold increase (95% CI: 1.94–4.86, P < 0.001) in dengue incidence rates among children aged 0–19 years [35]. Another study from Nepal, utilizing a Poisson generalized additive model revealed that a significant positive correlation between NDVI and dengue incidence at lag 0 and lag 1 period, with a stronger relationship observed at lag 0 period [36].

The findings of our study revealed a complex and evolving relationship between the Normalized Difference Water Index (NDWI) and dengue incidence in Nepal from 2020 to 2023. Specifically, this study observed a significant negative correlation between NDWI and dengue incidence in 2020 and 2021, with Moran’s I values of −0.177 and −0.128, respectively. This indicated that areas with higher water content, as captured by the NDWI, were associated with lower dengue transmission during those years. However, in 2023, the relationship shifted, showing a positive correlation with a Moran’s I value of 0.139, suggesting that higher water content was associated with increased dengue incidence. Notably, in 2022, the spatial relationship between NDWI and dengue was not statistically significant, highlighting the variability in the factors that influenced dengue transmission over time.

The significant negative correlation observed in 2020 and 2021 might be explained by the fact that high NDWI values typically indicated the presence of bodies of water or areas with higher moisture content, which could have disrupted mosquito breeding sites, particularly if the water sources were large or subject to heavy runoff. However, the shift to a positive correlation in 2023 suggested that changes in ecological or environmental factors may have created more favorable conditions for mosquito breeding in areas with higher water content, possibly due to the presence of stagnant water bodies or changes in land use or climate conditions. The lack of significant correlation in 2022 implied that NDWI alone was not a strong predictor of dengue incidence that year, indicating that other factors, such as variations in mosquito control efforts, human behavior, or climate anomalies, may have played a more critical role in determining dengue transmission patterns. These findings underscored the importance of considering the dynamic nature of environmental factors in dengue transmission and suggested that water-related interventions should be tailored to specific local conditions and adjusted over time as environmental conditions changed. They also highlighted the need for ongoing monitoring and the integration of NDWI with other environmental and demographic data to develop more accurate predictive models for dengue risk and to guide targeted interventions.

A study conducted in China using Ordinary Least Squares regression found a significant positive correlation between dengue fever outbreaks and the Normalized Difference Water Index (NDWI), with a correlation coefficient of 0.456. This finding aligns with our results from 2023 [11]. Additionally, a 2019 study from Bangladesh supports our 2023 findings, highlighting that region with higher NDWI scores experienced more severe dengue epidemics [37]. A study from Argentina using the MaxEnt predictive model found that environmental variables, particularly NDWI, accounted for 75% of the distribution of Ae. aegypti breeding sites, with water distribution being the most significant factor at 11.6% [38].

This study examined the relationship between Land Surface Temperature during the day (LSTD) and at night (LSTN) and the incidence of dengue. LSTD represents the surface temperature of the earth during the daytime, while LSTN reflects nighttime surface temperatures. Both temperature measures can influence the breeding and activity patterns of mosquitoes that carry dengue. Our analysis identified a negative correlation between LSTD density and dengue incidence, with Moran’s I values of −0.141 in 2020 and −0.174 in 2021, indicating significant spatial autocorrelation during these years. However, the data from 2022 and 2023 did not show any significant spatial patterns. Similarly, LSTN density was also negatively correlated with dengue incidence, with Moran’s I values of −0.187 in 2020 and −0.205 in 2021. Like LSTD, the relationship was not spatially significant in 2022 and 2023.

The findings suggested that lower land surface temperatures, both during the day and at night, were associated with higher rates of dengue transmission in 2020 and 2021. This inverse relationship may be due to the fact that cooler surface temperatures create more favourable microclimatic conditions for mosquito survival and breeding. Lower daytime temperatures might reduce evaporation rates, leading to more standing water, which serves as breeding grounds for mosquitoes. Additionally, lower nighttime temperatures could prolong the activity and survival of mosquitoes, thereby extending the period during which they can transmit the virus.

The absence of significant spatial patterns in 2022 and 2023 indicates that the relationship between land surface temperature and dengue incidence is not consistent over time, suggesting that other environmental or socio-economic factors may have had a greater impact on dengue transmission during these years. Factors such as changes in climate patterns, urbanization, public health interventions, or variations in mosquito control efforts could have influenced these results. These findings highlight the complexity of the factors influencing dengue transmission and underscore the importance of considering multiple environmental variables in predictive models. Understanding the interplay between temperature and mosquito behavior is crucial for developing more targeted and effective dengue prevention and control strategies.

Using Bayesian Poisson spatial analysis, a study from Indonesia supports our results by identifying the average minimum nighttime temperature as the only significant environmental risk factor for dengue infection. This study reported a 28% increase in dengue risk in areas with nighttime temperatures between 10°C and 15°C, and a 64% increase in areas with temperatures below 10°C, compared to areas with temperatures of 15°C or higher [39]. However, our findings contradicted previous studies from China [11] and two studies from Nepal [13,36]. These studies highlighted that there was a positive spatial correlation between Dengue incidence and nighttime LST density. One study reported a significant positive correlation (p < 0.05) between annual dengue cases and increases in average monthly minimum temperatures during the monsoon season without time lag (R2 = 0.67, p = 0.01). It emphasized that higher nighttime temperatures during the transmission season are crucial predictors of dengue risk [13]. Additionally, another study found no significant correlation between dengue and daytime LST (day LST) or nighttime LST (night LST) until lag 3. However, by lag 5, both day LST and night LST showed a positive association with dengue fever. The significant correlation with day LST persisted until lag 6, whereas night LST correlation was not significant during that period [36].

This present study utilized precipitation data from the CHIRPS Pentad dataset, known for its high resolution and temporal frequency, to explore the relationship between precipitation and dengue incidence. The analysis revealed an evolving pattern in the correlation between precipitation and dengue transmission over the study period. Initially, precipitation showed a negative correlation with dengue incidence, as indicated by Moran’s I values of −0.183 in 2020 and −0.159 in 2021. However, this relationship gradually shifted to a positive correlation in subsequent years, with Moran’s I values of 0.154 in 2022 and 0.225 in 2023. The shift from a negative to a positive correlation between precipitation and dengue incidence over the years highlights the complex and dynamic nature of the relationship between weather patterns and disease transmission. In 2020 and 2021, lower precipitation levels were associated with higher dengue incidence, possibly due to the fact that moderate rainfall can create ideal conditions for mosquito breeding by leaving behind small, stagnant water bodies. Excessive rainfall, on the other hand, may initially disrupt breeding sites by washing away larvae or by causing flooding, which reduces the availability of suitable habitats for mosquitoes.

As the correlation became positive in 2022 and 2023, the data suggested that increased precipitation contributed to higher dengue transmission. This could be due to a cumulative effect where consistent rainfall over time led to a proliferation of breeding sites, thus increasing the mosquito population and, consequently, the risk of dengue. The positive correlation in the latter years might also reflect changes in environmental or socio-economic factors, such as inadequate drainage systems or shifts in population density and movement, which could exacerbate the impact of increased precipitation on dengue transmission. These findings underscored the importance of considering temporal changes in weather patterns and their interaction with other factors when developing predictive models for dengue outbreaks. The evolving relationship between precipitation and dengue incidence suggested that adaptive and context-specific public health strategies are needed to mitigate the impact of dengue in regions with varying rainfall patterns. The strong positive spatial autocorrelation observed in 2022 indicates a concentrated outbreak, with urban areas like Kathmandu and Kaski showing significantly higher dengue incidence. The positive correlation between precipitation and dengue in 2022–2023 suggests that increased rainfall may provide favourable conditions for mosquito breeding, highlighting the importance of targeting mosquito control measures during rainy seasons.

These findings aligned with previous research. For instance, a study from Sri Lanka reported that average rainfall had a significant positive correlation with dengue incidence (p < 0.0001), indicating that high rainfall contributes to an increased incidence of dengue [40]. In Thailand, higher rainfall has been linked to increased dengue incidence. Specifically, a 1% increase in rainfall in Bangkok was associated with a 3.3% rise in dengue cases [41]. Similarly, in Khon Kaen province, northeastern Thailand, a 1 cm increase in monthly rainfall was associated with a 0.4% increase in dengue incidence [42]. A study from Nepal aligned with our findings, showing that the temporal pattern of dengue incidence followed the seasonality of monsoon rainfall examined that the temporal variability of dengue fever and its association with remotely sensed environmental variables in the Chitwan district of Nepal [43]. It also assessed the delayed effects of environmental variables on dengue transmission. The association between precipitation and dengue fever was significantly positive from lag 0 to lag 3 periods, with a particularly strong correlation at lag 1 period [43].

However, two studies from Indonesia presented conflicting findings with our results. One study suggested that an increase in rainfall is significantly associated with a slight decrease in childhood dengue incidence by 1% (P < 0.001) over the following two months [35]. Moreover, another study using the Fusion area-cell spatiotemporal generalized Geo-additive-Gaussian Markov random field (FGG-GMRF) model proposed that precipitation generally has a negative impact, with an estimated global mean effect of −0.0041. This implies that for every one mm increase in rainfall, dengue risk decreases by 0.41%. The rationale provided was that heavy rainfall disrupts the reproductive cycle of Aedes mosquitoes by washing away their breeding sites [43].

### Strengths and limitations

The strength of this study lies in its comprehensive analysis of dengue incidence across all 77 districts of Nepal, allowing for the identification of districts requiring targeted dengue interventions. However, the study is limited by its reliance on secondary data, which does not allow for the determination of causality. Additionally, the quality of the data is uncertain due to the lack of real-time information, which could result in underreporting, overreporting, or misdiagnosis of dengue cases.

Suggestions for future research include incorporating real-time data collection and validation to improve the accuracy and reliability of findings. Conducting primary studies to understand the underlying causes and factors influencing dengue incidence would also enhance the effectiveness of intervention strategies. Furthermore, to address gaps in the understanding of dengue epidemiology in Nepal underscore the need for community-based cohort studies across various regions to obtain reliable age-specific incidence data, as well as studies to generate dengue seroprevalence data nationwide.

## Conclusion

This study analyzed dengue incidence in Nepal from 2020 to 2023, uncovering significant variations in disease patterns and their interconnection to the environmental factors. The findings revealed a sharp escalation in dengue cases in 2022, followed by a declination in 2023, likely due to public health interventions. While no significant spatial patterns were observed in 2020 and 2021, a focused distribution of cases emerged in 2022 and 2023. This study underscores the importance of considering environmental factors such as precipitation and vegetation in dengue control efforts. Future research should focus on real-time monitoring of environmental variables to predict and manage outbreaks more effectively, especially in urban areas with high dengue risk.

The study also highlighted shifting correlations between dengue incidence and environmental factors like vegetation, water content, temperature, and precipitation over time. These results underscored the need for adaptive public health strategies to effectively manage dengue outbreaks, with further research recommended to refine predictive models and interventions.
